# Pharmacogenomics Study for Raloxifene in Postmenopausal Female with Osteoporosis

**DOI:** 10.1155/2020/8855423

**Published:** 2020-08-31

**Authors:** Hsing-Fang Lu, Po-Hsin Chou, Gan-Hong Lin, Wan-Hsuan Chou, Shih-Tien Wang, Wirawan Adikusuma, Eko Mugiyanto, Kuo-Sheng Hung, Wei-Chiao Chang

**Affiliations:** ^1^Department of Clinical Pharmacy, School of Pharmacy, Taipei Medical University, Taipei, Taiwan; ^2^School of Medicine, National Yang-Ming University, Taipei, Taiwan; ^3^Department of Orthopedics and Traumatology, Taipei Veterans General Hospital, Taipei, Taiwan; ^4^Clinical Pharmacogenomics and Pharmacoproteomics, School of Pharmacy, Taipei Medical University, Taipei, Taiwan; ^5^Department of Pharmacy, University of Muhammadiyah Mataram, Mataram, Indonesia; ^6^Clinical Drug Development of Chinese Herbal Medicine, School of Pharmacy, Taipei Medical University, Taipei, Taiwan; ^7^Graduate Institute of Injury Prevention and Control, College of Public Health, Taipei Medical University, Taipei 11042, Taiwan; ^8^Department of Neurosurgery, Taipei Medical University-Wan Fang Hospital, Taipei, Taiwan; ^9^Pain Research Center, Wan Fang Hospital, Taipei Medical University, Taipei, Taiwan; ^10^Biotechnology Research and Development, College of Pharmacy, Taipei Medical University, Taipei, Taiwan

## Abstract

Osteoporosis is characterized by decreased bone mineral density and increased risk of fracture. Raloxifene is one of the treatments of osteoporosis. However, the responses were variable among patients. Previous studies revealed that the genetic variants are involved in the regulation of treatment outcomes. To date, studies that evaluate the influence of genes across all genome on the raloxifene treatment response are still limited. In this study, a total of 41 postmenopausal osteoporosis patients under regular raloxifene treatment were included. Gene-based analysis using MAGMA was applied to investigate the genetic association with the bone mineral density response to raloxifene at the lumbar spine or femoral neck site. Results from gene-based analysis indicated several genes (*GHRHR*, *ABHD8*, and *TMPRSS6*) related to the responses of raloxifene. Besides, the pathways of iron ion homeostasis, osteoblast differentiation, and platelet morphogenesis were enriched which implies that these pathways might be relatively susceptible to raloxifene treatment outcome. Our study provided a novel insight into the response to raloxifene.

## 1. Introduction

Osteoporosis is defined as the weakened architecture of the bone and elevated risk of fracture [1]. In Taiwan, the prevalence of osteoporosis is about 6.9% in males over the age of 65 and 21.2% in postmenopausal females [2]. Several risk factors of osteoporosis have been identified, including menopause, dairy intake, life style, and genetic factors [3]. Osteoporosis is a polygenic disease that is determined by multiple genetic effects from several genes. Variants near *WNT16* and *FONG* have been reported to be associate with bone mineral density (BMD) [4–7]. The risk of osteoporosis increases dramatically in the elderly population, that bone fractures are known to increase mortality and lead to an enormous healthcare burden on society [8, 9]. Since the aged population is growing rapidly, osteoporosis has become a crucial clinical issue.

Nonsteroidal selective estrogen-receptor modulators (SERMs) can inhibit bone resorption. SERMs have been widely used in the prevention and treatment of postmenopausal osteoporosis [10]. Raloxifene is a SERM agent used in the clinic for postmenopausal osteoporosis [11]. The drug is a partial agonist for the estrogen receptor and is known to inhibit proliferation of breast epithelium, while preserving bone mineral density (BMD). This drug increases BMD and reduces the occurrences of new vertebral fractures [12]. However, the therapeutic response of raloxifene varies widely among patients. Previous studies revealed that two polymorphisms of *ESR*1 (estrogen receptor 1) (rs9340799 and rs2234693) are associated with the BMD response to raloxifene in osteoporosis patients [13, 14]. UGT1A8 (UDP glucuronosyltransferase family 1 member A8) encodes an enzyme involved in extrahepatic glucuronidation of raloxifene [15], and UGT1A8∗2 was found to be associated with more raloxifene glucuronide metabolite formation in human jejunum tissue [16]. Labad et al. also identified a missense variant of *UGT1A8*, rs1042597, that associated with the treatment responses to raloxifene in postmenopausal schizophrenic women [17].

Previous studies focused on the association between therapeutic responses and variants of a single gene. This type of study design called a candidate gene approach, which is a hypothesis-driven method that tests a small number of variants [18]. Limited genetic variants are unable to discover novel mechanisms. On the other hand, genome-wide association study (GWAS) is a hypothesis-free or hypothesis-generating method that provides opportunities to identify novel insights into the phenotype. In addition, GWAS is able to explore new genetic factors of complex human traits [19]. Since the mechanism of how genes influence response of raloxifene remains unclear, the aim of the current study is using gene-based analysis to identify the main network correlated with the responses of raloxifene.

## 2. Materials and Methods

### 2.1. Sample Collection

We collected samples from the neurosurgery outpatient clinic in Wang-Fang Hospital, Taipei, Taiwan. Our inclusion criteria were as follows: (1) 60 to 85-year-old postmenopausal female, (2) *T*‐score≦−2.5, and (3) under regular raloxifene treatment (60 mg/day). Patients with continuous steroid use (of over 6 months) or long-term inflammatory disease were excluded. In total, 41 osteoporosis patients were recruited. Bone mineral density (BMD) was measured by dual-energy radiograph absorptiometry (Lunar Prodigy, version 9.1, GE Healthcare, Madison, WI, USA) with standard protocols at the lumbar spine (LS; L2-4 or L1-4) and femoral neck (FN). Age, gender, body mass index (BMI), medication profile, and follow-up duration of all individual were collected. We also collected the BMD profiles after patients receiving treatment for 1-2 years. The study was approved by the Joint Institutional Review Board of Taipei Medical University. All subjects provided written informed consent.

### 2.2. DNA Extraction and Genotyping Array

Peripheral whole blood was collected from the patients. Gentra Puregene Blood Kit (Qiagen, Valencia, CA, USA) was used to extract the genomic DNA. The extraction process was according to the user manual of the product. The quality of DNA was confirmed by 1% agarose gel electrophoresis. Sample with no obvious degradation can be submitted to further analysis. Affymetrix Axiom TWB SNP Array (Thermo Fisher Scientific, USA) was used to perform GWAS. The process was done by the National Center for Genome Medicine, Taiwan (NCGM).

### 2.3. Quality Control

PLINK [20] was applied to conduct the quality control of genotype data. We excluded SNPs with low genotyping call rate (<0.95), minor allele frequency less than 0.05, and *P* value of Hardy-Weinberg equilibrium less than 1 × 10^−6^. Individuals with a sample call rate less than 0.95 were excluded. The imputation task was done by Michigan Imputation Server (Minimac3). Data from the Haplotype Reference Consortium (HRC) was used as reference panels for imputation in order to increase statistical power. After imputation, only variants which MAF≧0.05 and *R*^2^≧0.3 were included in the association analysis. Principle components (PCs) were generated by PLINK with samples from HapMap3. A linear regression model was used for statistically testing the associations between SNPs and phenotypes by *PLINK2*. We defined the percentage change of bone mineral density from baseline by the following equation:

(1) $$\begin{array}{c} x=\frac{2^{\text{nd}}\text{BMD}-1^{\text{st}}\text{BMD}}{1^{\text{st}}\;BMD}. \end{array}$$

We further normalized phenotype before association analysis. We included age, BMI, follow-up months, and PC1 to PC10 as covariates in the analysis model. The Manhattan plot and qq plot were visualized by the R package “qqman,” and the suggestive significant threshold was 1 × 10^−5^ as the default setting of this package. The regional plots were generated by LocusZoom [21].

### 2.4. Variant Annotation

We annotated the rs number of candidate variates by ANNOVAR [22] with avsnp150 database. The annotation of nearby genes was according to the NCBI RefSeq hg19 reference panel. In order to compare our results with previous studies, the data of GWAS catalog v1.0.2 (20190322) was downloaded from GWAS catalog website.

### 2.5. Gene-Based Analysis and Gene-Set Analysis

Since our sample size was very limited, the effects of individual markers may be too weak to detect. Therefore, we applied MAGMA to conduct gene-based analysis to increase the power [23]. This method can analyze multiple genetic markers together to determine their joint effect. Input SNPs were mapped to 18306 protein coding genes; therefore, the Bonferroni significant threshold for gene analysis was using *P* < 2.73 × 10^−6^. To understand the biological enriched pathways related with the raloxifene treatment response, we used gene-set analysis by MAGMA. The curated gene sets and GO terms were obtained from MsigDB v6.1. Curated gene sets were collected from 9 data resources including KEGG, Reactome, BioCarta, and biomedical literatures. Variates located within transcription start region and stop region of the gene region will be mapped to that gene. We used 1000 genomes phase 3 as reference genome.

## 3. Results

### 3.1. Baseline Characteristics

**Figure 1:**
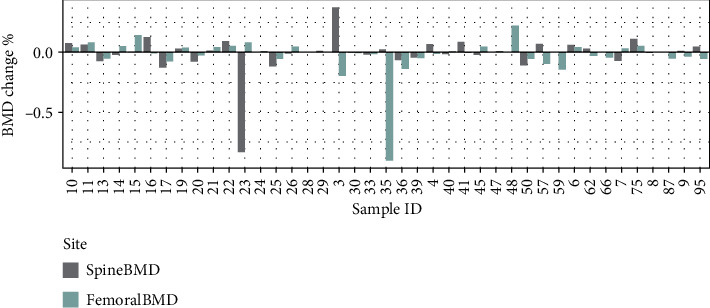
The distribution of the BMD change % at the lumbar spine and femoral neck site.

The distribution of the BMD change percent is shown in Figure 1. After treatment, the BMD response to raloxifene was diverse at both lumbar spine and femoral neck sites in patients. In total, 41 patients were assessed for femoral neck BMD (S Table 1), and the average percentage of BMD change was −2.5 ± 15.8%. The mean follow-up time was 35.61 ± 23.54 months among all subjects. The mean age of subjects was 72.4 ± 7.0 years, and the average BMI was 23.96 ± 3.49 kg/m^2^. Among the 41 patients assessed for FN BMD, 34 were also assessed for lumbar spine BMD. The average age and BMI of this subset were similar to the complete group.

### 3.2. Association Analysis

**Figure 2:**
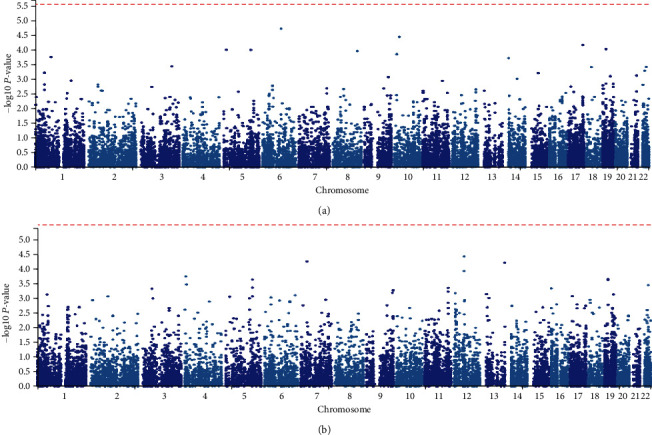
Manhattan plot of the gene-based test based on response to raloxifene. The red line represented suggestive significant threshold (*P* = 2.73 × 10^−6^): (a) Lumbar spine site and (b) Femoral neck site.

We conducted a genome-wide association analysis to reveal the risk loci associated with raloxifene treatment response. A principal component analysis showed that there was no population stratification (S Figure 1). Neither site revealed genome-wide significant association signal of BMD change (S Figures 2, 3). However, there were 112 variants with suggestive significance for LS percent BMD change, while 13 variants showed significance for FN percentage BMD change. The most significant variant in the LS site was rs7768089 (*P* = 2.29 × 10^−6^), which was an intronic SNP of *FUT9* located on 6q16.1. Meanwhile, rs34311394 at 12q21.2 was the most significant SNP (*P* = 4.44 × 10^−6^) for the FN site. This SNP is located in the intergenic region between *CSRP2* and *E2R7*. There was no overlap variants among suggestively significant SNPs between the two sites.

### 3.3. Candidate Genes Cause Decreased BMD in Mutant Mice

Next, a gene-based analysis was conducted by MAGMA. However, none of the genes reached the threshold of statistical significance (Figure 2). The top ten significant genes at the LS and FN sites are shown in Tables 1 and 2. To understand the potential biological functions of these genes, we queried the phenotypes of mutant mice in the International Mouse Phenotype Consortium (IMPC) database. Among the queried genes, *GHRHR*, *ABHD8*, and *TMPRSS6* showed decreased BMD in a homozygous mutant mouse in both sexes.

### 3.4. Enrichment of Osteoblast Differentiation Pathway

We further applied gene-set analysis to reveal the significant enriched gene sets. After FDR adjustment, although none of the pathway achieved the statistical significance (Tables 3 and 4), iron homeostasis for the LS site (*P* = 2 × 10^−4^) was the most significant gene set. Regarding the femoral neck site, the most significant gene set was platelet morphogenesis (*P* = 1 × 10^−4^). In addition, another gene sets of “osteoblast differentiation by phenylamil up” showed nominal significance for the femoral neck site as well (*P* = 4 × 10^−4^).

### 3.5. Comparison with Previous Candidate Variants

Since some variants have been reported to be associated with response to raloxifene, we next focused two SNPs within *ESR1* (rs9340799 and rs2234693) and one SNP in *UGT1A8* (rs1042597). As shown in Supplementary Table 2, none of the SNPs demonstrated nominal significance (*P* > 0.05) at the LS or FN site. We further extracted larger regions around these two genes (±500 Mb) of two phenotypes and generated regional plots. However, no variant within these regions reached suggestive significance (S Figure 4-7).

## 4. Discussion

This study used two skeletal positions to assess the relationships between BMD response to raloxifene and gene network. Although statistical power was limited sample size, several candidates including *GHRHR*, *ABHD8*, and *TMPRSS6* still addressed significance after gene-based analysis. These three genes were also associated with decreased BMD in mutant mouse models. From gene-set analysis, we found that iron homeostasis and osteoblast differentiation gene sets were enriched. Previous studies indicated that iron overload affects the bone phenotype and increases risk of bone fracture. Iron overload is an important factor that is involved in the activation of osteoclast, the differentiation process, and the reduction of osteoblast formation [24, 25]. Besides, in patients with thalassemia major, which is often characterized by low BMD values and iron overload, the circulating IGF-1 levels and its binding protein are lower than those in the healthy individuals [26]. Thus, IGF-1 is critical in bone remodeling. The lower level of IGF-1 may increase the risk of hip fracture [27]. Taken together, iron overload may increase bone resorption and decrease bone formation through osteoblast and osteoclast activity or through the endocrine growth hormone- (GH-) IGF-1 axis pathway.

Heilberg et al. reported that the rs9340799 (Xbal) AA genotype and rs2234693 (PvuII) CC genotype of the *ESR1* have higher LS bone density after one-year treatment of raloxifene in Hispanic women [14]. Conversely, Mondockova et al. revealed that the AA genotype of rs9340799 and TT genotype of rs2234693 showed worse treatment response to raloxifene at the LS site in Slovak women [13]. Because of the inconsistent findings, we tried to confirm it by using this study. However, none of the variants were associated with the response to raloxifene in the current study. In another study, individuals with the CC genotype of the missense SNP (Ala173Gly) of *UGT1A8* revealed more improvement in negative symptoms after raloxifene treatment [17]. However, this variant cannot be replicated in the current study as well. We found that the most significant association with drug response at LS site was an intronic SNP of fucosyltransferase 9 (*FUT9*), rs7768089. This gene is a member of the glycosyltransferase family, and it participates in the biosynthesis of Lewis antigen. HaploReg v4.1 demonstrated that rs7768089 may alter the transcription factor (Foxa disc2, Nrd-2 3, and TCF11::MafG) binding affinity, and according to GTEx, this SNP is an expression quantitative locus (eQTL) of *FUT9* in the pancreas and brain tissue (S Table 3). However, the mechanistic relationship between *FUT9* and BMD remains to be clarified.

For the FN site, the most significant SNP (rs34311394) was located within the intron between *CSRP2* and *E2F7*. *CSRP2* (cysteine and glycine rich protein 2) is involved in development and cellular differentiation, and E2F7 (E2F transcription factors) participates in the regulation of cell cycle progression. This SNP is associated with the expression of *CSRP2* in the brain tissue (S Table 3). The most significant association revealed by a gene-based study was *GHRHR* (growth hormone-releasing hormone receptor). Although no traits related to BMD or drug response have been reported, the Ghrhr^tm1.1(KOMP)Vlcg^ mouse model showed decreased bone mineral density according to the IMPC database. Based on the results in GWAS catalog, the genetic variants of *GHRHR* associated with congenital left sided heart lesions and adolescent idiopathic scoliosis. Further functional studies are needed to determine the plausible impact of these candidate genes on the bone.

Due to its estrogenic effects, raloxifene may increase the risk of thromboembolic events, such as deep vein thrombosis and pulmonary embolism. Estrogen increases the concentration of plasma fibrinogen and coagulation factors and enhances the aggregation of platelets [28]. From the gene-set analysis for the femoral neck site, the most significant treatment-associated gene-set was platelet morphogenesis. These results hint that genetic variants could possibly play a role in the occurrence of side effects. Thus, further studies may focus on the pharmacogenomics of thromboembolic events in raloxifene users. The main limitation of the current study is the sample size which is not able to confer sufficient statistical power to reach genome-wide significance. Larger cohorts are needed to replicate our results. Another limitation is the in the study. The majority of subjects are elderly patients with a mean age of 72.4 years. Previous studies have reported that bone loss may accelerate in the individuals who are over 70 years old [29]. In addition, aging influences GH and IGF-1 serum levels [27], which might result in the change of BMD levels and the pharmacological effects. Further studies should also consider the age, the concentration of raloxifene in the blood, and the drug compliance for each patient.

## 5. Conclusions

In summary, we found plausible gene sets in iron ion homeostasis, osteoblast differentiation, and platelet morphogenesis pathways that may influence raloxifene responses. These findings offer new important insights and warrant further investigations into the pharmacogenomics of raloxifene.

## Figures and Tables

**Table 1:** Top 10 significant genes of gene-based analysis at the lumbar spine site.

Gene	CHR	START	STOP	Number of SNPs	ZSTAT	*p* value	KO mouse bone phenotype∗
*FUT9*	6	96463860	96663488	474	4.11	2.0.*E* − 05	NA
*APBB1IP*	10	26727132	26856732	304	3.962	3.7.*E* − 05	NA
*MYO15B*	17	73584139	73622929	104	3.808	7.0.*E* − 05	NA
*USHBP1*	19	17359985	17393595	54	3.728	9.7.*E* − 05	Vertebral fusion
*DNAH5*	5	13690440	13944652	691	3.712	1.0.*E* − 04	No
*NRG2*	5	139226364	139422884	238	3.709	1.0.*E* − 04	NA
*KIAA0196*	8	126036502	126104082	211	3.691	1.1.*E* − 04	NA
*PHYH*	10	13319796	13344412	70	3.625	1.4.*E* − 04	No
*ELTD1*	1	79355449	79472403	309	3.567	1.8.*E* − 04	NA
*OR4K1*	14	20403767	20404842	7	3.545	2.0.*E* − 04	NA

CHR: chromosome; START: start position of the gene; STOP: stop position of the gene. ∗Knockout mouse bone-related phenotypes were queried from International Mouse Phenotype Consortium.

**Table 2:** Top 10 significant genes of gene-based analysis at the femoral neck site.

Gene	CHR	START	STOP	Number of SNPs	ZSTAT	*p* value	KO mouse bone phenotype∗
*AC068987.1*	12	52203789	52206636	1	3.953	3.90*E* − 05	NA
*GHRHR*	7	30978284	31032869	139	3.859	5.70*E* − 05	BMD decrease
*F10*	13	113777128	113803843	35	3.836	6.20*E* − 05	NA
*SCN8A*	12	51984050	52206648	200	3.674	1.20*E* − 04	NA
*TMEM128*	4	4237269	4249950	84	3.562	1.80*E* − 04	NA
*MRPL34*	19	17403418	17417652	35	3.51	2.20*E* − 04	NA
*HSPA9*	5	137890571	137911133	14	3.501	2.30*E* − 04	NA
*ABHD8*	19	17402940	17421045	42	3.494	2.40*E* − 04	BMD decrease
*ABLIM2*	4	7967039	8160559	488	3.394	3.40*E* − 04	NA
*TMPRSS6*	22	37461476	37505603	97	3.38	3.60*E* − 04	BMD decrease

CHR: chromosome; START: start position of the gene; STOP: stop position of the gene. ∗Knockout mouse bone-related phenotypes were queried from International Mouse Phenotype Consortium.

**Table 3:** Gene-set analysis of raloxifene response at lumbar spine site.

Database	Pathway	Number of genes	Beta	SE	*p* value	*q* value
GO bp	Iron ion homeostasis	63	0.363	0.101	2.00*E* − 04	0.884
GO bp	Regulation of immunoglobulin secretion	14	0.774	0.227	3.00*E* − 04	0.884
GO bp	Transition metal ion homeostasis	96	0.267	0.08	4.00*E* − 04	0.884
Curated gene sets	Inhibition of replication initiation of damaged DNA by rb1 e2f1	10	0.879	0.266	5.00*E* − 04	0.884
Curated gene sets	Apoptosis	82	0.28	0.086	6.00*E* − 04	0.884
GO bp	Response to protozoan	20	0.569	0.177	6.00*E* − 04	0.884
Curated gene sets	Cdh1 targets 3 dn	58	0.331	0.106	9.00*E* − 04	0.884
Curated gene sets	Treating iron overload	7	0.879	0.283	9.00*E* − 04	0.884
GO bp	Toll like receptor signaling pathway	77	0.3	0.099	1.00*E* − 03	0.884
GO bp	Cellular iron ion homeostasis	40	0.374	0.124	1.00*E* − 03	0.884
Curated gene sets	Tlx targets 60 hr dn	258	0.148	0.049	1.00*E* − 03	0.884
GO bp	Negative regulation of platelet activation	17	0.626	0.211	2.00*E* − 03	0.884
Curated gene sets	Tgfb1 signaling via nfic 10 hr dn	29	0.464	0.158	2.00*E* − 03	0.884
Curated gene sets	Response to tosedostat 24 hr dn	936	0.074	0.025	2.00*E* − 03	0.884
Curated gene sets	Metabolism of amino acids and derivatives	186	0.166	0.057	2.00*E* − 03	0.884
GO bp	Cellular defense response	54	0.323	0.111	2.00*E* − 03	0.884
Curated gene sets	Akt phosphorylates targets in the cytosol	12	0.559	0.193	2.00*E* − 03	0.884
Curated gene sets	Asbcell pathway	9	0.783	0.271	2.00*E* − 03	0.884
Curated gene sets	Cell cycle s	152	0.193	0.068	2.00*E* − 03	0.884
Curated gene sets	Ar tf pathway	47	0.352	0.124	2.00*E* − 03	0.884

GO: gene ontology; bp: biological process; *q* values were from false discovery rate.

**Table 4:** Gene-set analysis of raloxifene response at femoral neck site.

Database	Pathway	Number of genes	Beta	SE	*p* value	*q* value
GO bp	Platelet morphogenesis	16	0.762	0.208	1.00*E* − 04	0.859
GO bp	Regulation of nuclear transcribed mrna catabolic process deadenylation-dependent decay	14	0.633	0.187	3.00*E* − 04	0.859
GO bp	Cellular modified amino acid catabolic process	17	0.616	0.185	4.00*E* − 04	0.859
Curated gene sets	Osteoblast differentiation by phenylamil up	12	0.779	0.234	4.00*E* − 04	0.859
GO bp	Regulation of pri mirna transcription from rna polymerase ii promoter	16	0.691	0.209	5.00*E* − 04	0.859
GO bp	Regulation of establishment of protein localization to mitochondrion	118	0.234	0.073	7.00*E* − 04	0.966
GO bp	Regulation of protein targeting to mitochondrion	89	0.266	0.083	7.00*E* − 04	0.966
Curated gene sets	Werner syndrome and normal aging up	91	0.251	0.08	9.00*E* − 04	0.991
Curated gene sets	Aml methylation cluster 6 dn	32	0.418	0.137	1.00*E* − 03	0.999
GO bp	Epithelial structure maintenance	21	0.559	0.184	1.00*E* − 03	0.999
GO bp	Regulation of substrate adhesion-dependent cell spreading	41	0.379	0.129	2.00*E* − 03	0.999
Curated gene sets	Proteolytic cleavage of snare complex proteins	16	0.752	0.26	2.00*E* − 03	0.999
GO bp	Cardiac myofibril assembly	16	0.574	0.204	2.00*E* − 03	0.999
GO bp	Positive regulation of pri mirna transcription from rna polymerase ii promoter	10	0.702	0.25	2.00*E* − 03	0.999
Curated gene sets	Botulinum neurotoxicity	18	0.674	0.241	3.00*E* − 03	0.999
GO bp	Negative chemotaxis	38	0.391	0.142	3.00*E* − 03	0.999
GO bp	Regulation of endocrine process	44	0.348	0.126	3.00*E* − 03	0.999
Curated gene sets	Chondroitin sulfate biosynthesis	17	0.507	0.184	3.00*E* − 03	0.999
GO bp	Cellular metabolic compound salvage	35	0.36	0.131	3.00*E* − 03	0.999
GO bp	Cytokinesis	80	0.246	0.09	3.00*E* − 03	0.999

GO: gene ontology; bp: biological process; *q* values were from false discovery rate.

## Data Availability

The data used to support the findings of this study are included within the article.

## Supplementary Materials

Supplementary Materials Figure S1: evaluation of population stratification by principal component analysis. Figure S2: genome-wide association of response to raloxifene at lumbar spine bone mineral density. Figure S3: genome-wide association of response to raloxifene at femoral neck bone mineral density. Figure S4: *P* value of *ESR*region ± 500 Mb from response to raloxifene at LS site GWAS. Figure S5: *P* value of *UGT1A8*region ± 500 Mb from response to raloxifene at LS site GWAS. Figure S6: *P* value of ESR region ± 500 Mb from response to raloxifene at FN site GWAS. Figure S7: *P* value of *UGT1A8*region ± 500 Mb from response to raloxifene at FN site GWAS. Table S1: baseline characteristics of inclusion individuals. Table S2: variants reported to be related to raloxifene response previously Table S3: eQTL evidence of most significant SNPs.

## References

[1] Lorentzon M., Cummings S. R. (2015). Osteoporosis: the evolution of a diagnosis. *Journal of Internal Medicine*.

[2] Chen P. H., Lin M. S., Huang T. J., Chen M. Y. (2017). Prevalence of and factors associated with adopting bone health promoting behaviours among people with osteoporosis in Taiwan: a cross-sectional study. *BMJ Open*.

[3] Hendrickx G., Boudin E., Van Hul W. (2015). A look behind the scenes: the risk and pathogenesis of primary osteoporosis. *Nature Reviews Rheumatology*.

[4] Estrada K., Styrkarsdottir U., Evangelou E. (2012). Genome-wide meta-analysis identifies 56 bone mineral density loci and reveals 14 loci associated with risk of fracture. *Nature Genetics*.

[5] Lu H.‐. F., Hung K.-S., Chu H.-W. (2019). Meta-analysis of genome-wide association studies identifies three loci associated with stiffness index of the calcaneus. *Journal of Bone and Mineral Research*.

[6] Kou I., Takahashi A., Urano T. (2011). Common variants in a novel gene, FONG on chromosome 2q33.1 confer risk of osteoporosis in Japanese. *PLoS One*.

[7] Lu H.-F., Hung K.-S., Hsu Y.-W. (2015). Association study between the FTCDNL1 (FONG) and susceptibility to osteoporosis. *PLoS One*.

[8] Wright N. C., Looker A. C., Saag K. G. (2014). The recent prevalence of osteoporosis and low bone mass in the United States based on bone mineral density at the femoral neck or lumbar spine. *Journal of Bone and Mineral Research*.

[9] Weycker D., Li X., Barron R., Bornheimer R., Chandler D. (2016). Hospitalizations for osteoporosis-related fractures: economic costs and clinical outcomes. *Bone Reports*.

[10] Tella S. H., Gallagher J. C. (2014). Prevention and treatment of postmenopausal osteoporosis. *The Journal of Steroid Biochemistry and Molecular Biology*.

[11] Cummings S. R., Eckert S., Krueger K. A. (1999). The effect of raloxifene on risk of breast cancer in postmenopausal women. *JAMA*.

[12] Delmas P. D., Bjarnason N. H., Mitlak B. H. (1997). Effects of raloxifene on bone mineral density, serum cholesterol concentrations, and uterine endometrium in postmenopausal women. *New England Journal of Medicine*.

[13] Mondockova V., Adamkovicova M., Lukacova M. (2018). The estrogen receptor 1 gene affects bone mineral density and osteoporosis treatment efficiency in Slovak postmenopausal women. *BMC Medical Genetics*.

[14] Heilberg I. P., Hernandez E., Alonzo E. (2009). Estrogen receptor (ER) gene polymorphism may predict the bone mineral density response to raloxifene in postmenopausal women on chronic hemodialysis. *Renal Failure*.

[15] Kemp D. C., Fan P. W., Stevens J. C. (2002). Characterization of raloxifene glucuronidation in vitro: contribution of intestinal metabolism to presystemic clearance. *Drug Metabolism and Disposition*.

[16] Sun D., Jones N. R., Manni A., Lazarus P. (2013). Characterization of raloxifene glucuronidation: potential role of UGT1A8 genotype on raloxifene metabolism in vivo. *Cancer Prevention Research*.

[17] Labad J., Martorell L., Huerta-Ramos E. (2016). Pharmacogenetic study of the effects of raloxifene on negative symptoms of postmenopausal women with schizophrenia: a double-blind, randomized, placebo-controlled trial. *European Neuropsychopharmacology*.

[18] Kwon J. M., Goate A. M. (2000). The candidate gene approach. *Alcohol Research & Health*.

[19] Duncan L. E., Ostacher M., Ballon J. (2019). How genome-wide association studies (GWAS) made traditional candidate gene studies obsolete. *Neuropsychopharmacology*.

[20] Purcell S., Neale B., Todd-Brown K. (2007). PLINK: a tool set for whole-genome association and population-based linkage analyses. *American Journal of Human Genetics*.

[21] Pruim R. J., Welch R. P., Sanna S. (2010). LocusZoom: regional visualization of genome-wide association scan results. *Bioinformatics*.

[22] Wang K., Li M., Hakonarson H. (2010). ANNOVAR: functional annotation of genetic variants from high-throughput sequencing data. *Nucleic Acids Research*.

[23] de Leeuw C. A., Mooij J. M., Heskes T., Posthuma D. (2015). MAGMA: generalized gene-set analysis of GWAS data. *PLoS Computational Biology*.

[24] Balogh E., Paragh G., Jeney V. (2018). Influence of iron on bone homeostasis. *Pharmaceuticals*.

[25] Jeney V. (2017). Clinical impact and cellular mechanisms of Iron overload-associated bone loss. *Frontiers in Pharmacology*.

[26] Gaudio A., Morabito N., Catalano A., Rapisarda R., Xourafa A., Lasco A. (2019). Pathogenesis of thalassemia major-associated osteoporosis: a review with insights from clinical experience. *Journal of Clinical Research in Pediatric Endocrinology*.

[27] Geusens P. P. M. M., Boonen S. (2002). Osteoporosis and the growth hormone-insulin-like growth factor axis. *Hormone Research*.

[28] Bonnar J. (1987). Coagulation effects of oral contraception. *American Journal of Obstetrics and Gynecology*.

[29] Berger C., Langsetmo L., Joseph L. (2008). Change in bone mineral density as a function of age in women and men and association with the use of antiresorptive agents. *CMAJ*.

