# Supplementary material for: Pharmacogenomics Study for Raloxifene in Postmenopausal Female with Osteoporosis
Source: Dis Markers. 2020 Aug 31;2020:8855423. doi: 10.1155/2020/8855423 (PMC7479487; doi:10.1155/2020/8855423)
Supplement: Supplementary Materials — Figure S1: evaluation of population stratification by principal component analysis. Figure S2: genome-wide association of response to raloxifene at lumbar spine bone mineral density. Figure S3: genome-wide association of response to raloxifene at femoral neck bone mineral density. Figure S4: P value of ESRregion ± 500 Mb from response to raloxifene at LS site GWAS. Figure S5: P value of UGT1A8region ± 500 Mb from response to raloxifene at LS site GWAS. Figure S6: P value of ESR region ± 500 Mb from response to raloxifene at FN site GWAS. Figure S7: P value of UGT1A8region ± 500 Mb from response to raloxifene at FN site GWAS. Table S1: baseline characteristics of inclusion individuals. Table S2: variants reported to be related to raloxifene response previously Table S3: eQTL evidence of most significant SNPs. [file 8855423.f1.docx]

**Supplementary**

| **A** | **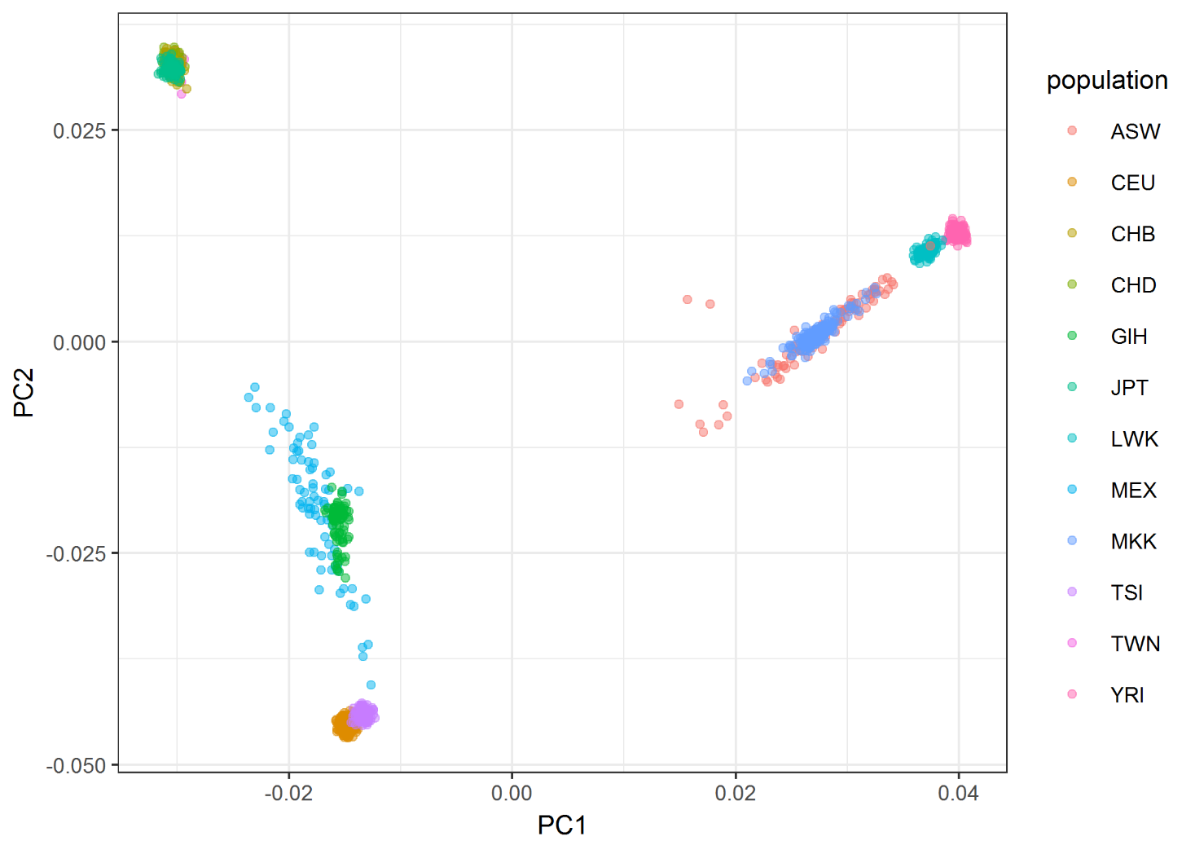** |
| --- | --- |
| **B** | **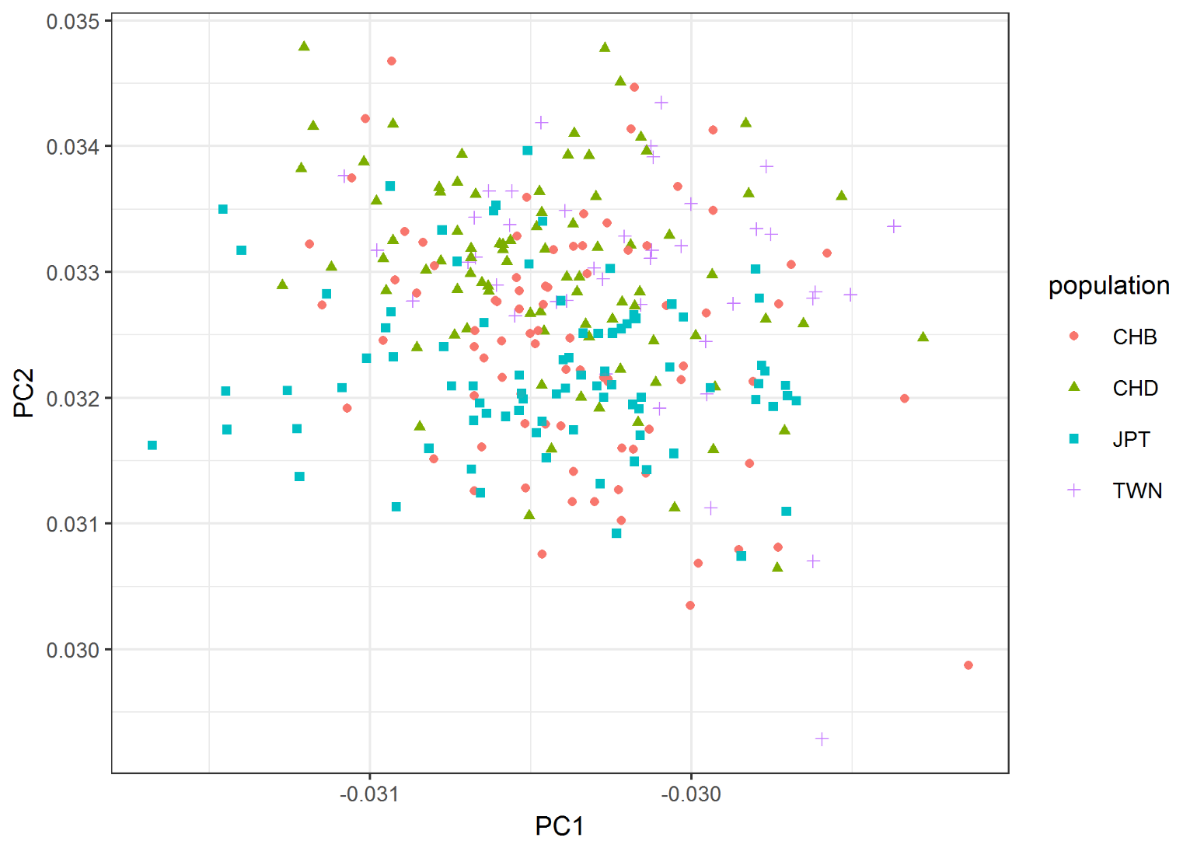** |

**Figure S1. Evaluation of population stratification by principle component analysis.** Figures illustrated the first two principle components based on analysis. (A) PCA of our study and HapMap. (B) PCA of our study and East Asia. TWN: Taiwanese.

**
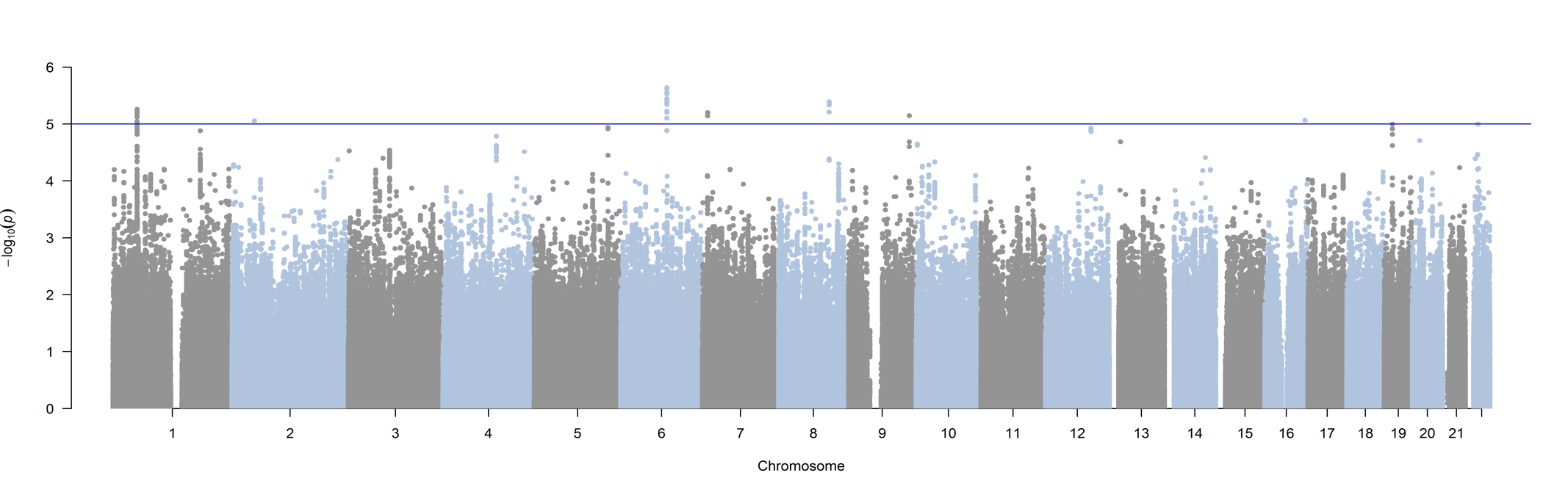
**

**Figure S2. Genome-wide association of response to raloxifene at lumbar spine bone mineral density.** The blue line represented suggestive significant threshold (1 x 10^-5^).

**
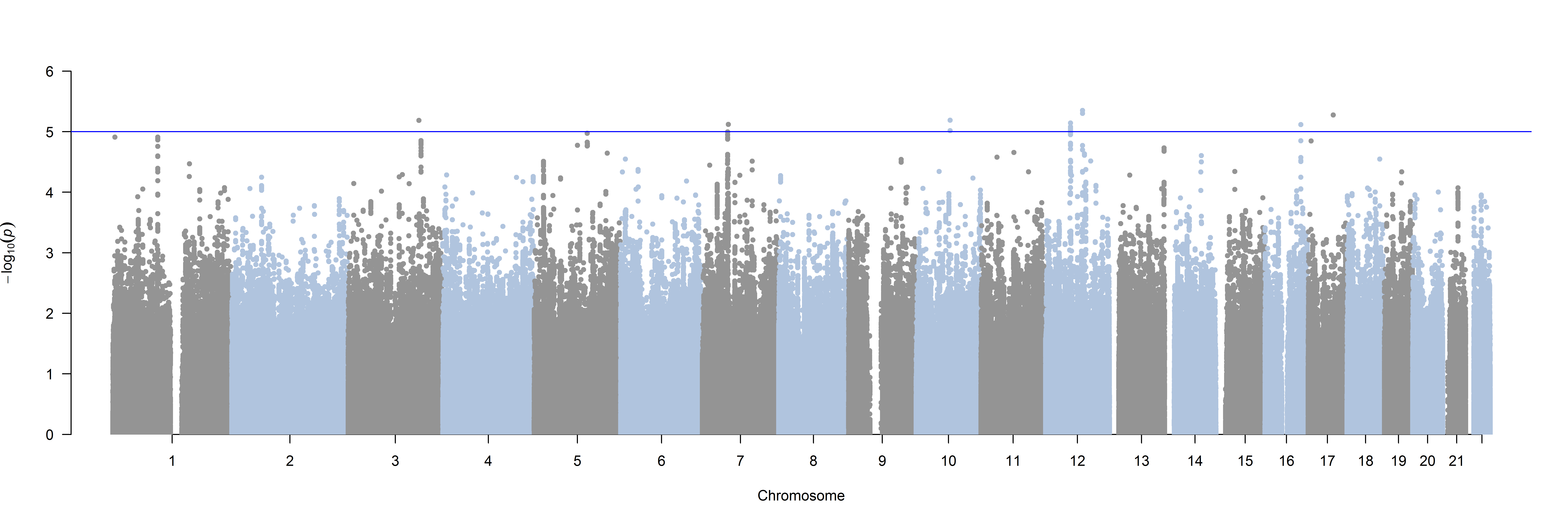
**

**Figure S3. Genome-wide association of response to raloxifene at femoral neck bone mineral density.** The blue line represented suggestive significant threshold (1 x 10^-5^).


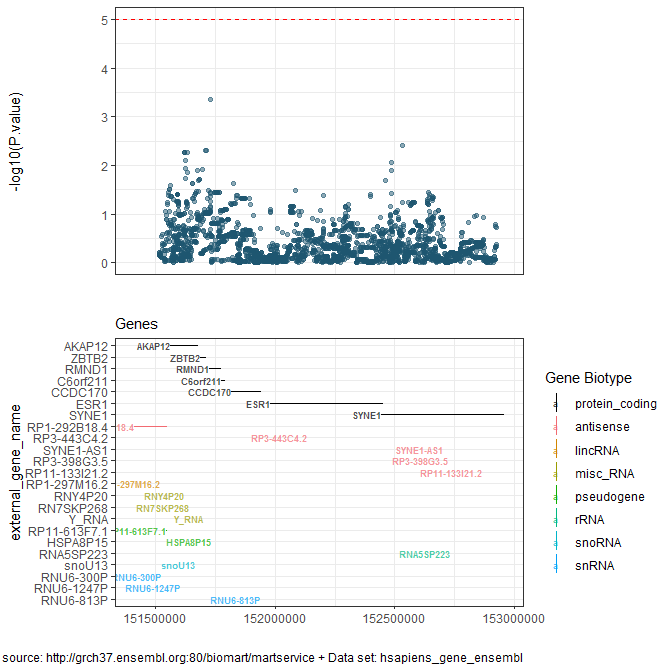


**Figure S4.　P value of *ESR* region ± 500 Mb from response to raloxifene at LS site GWAS.**

**
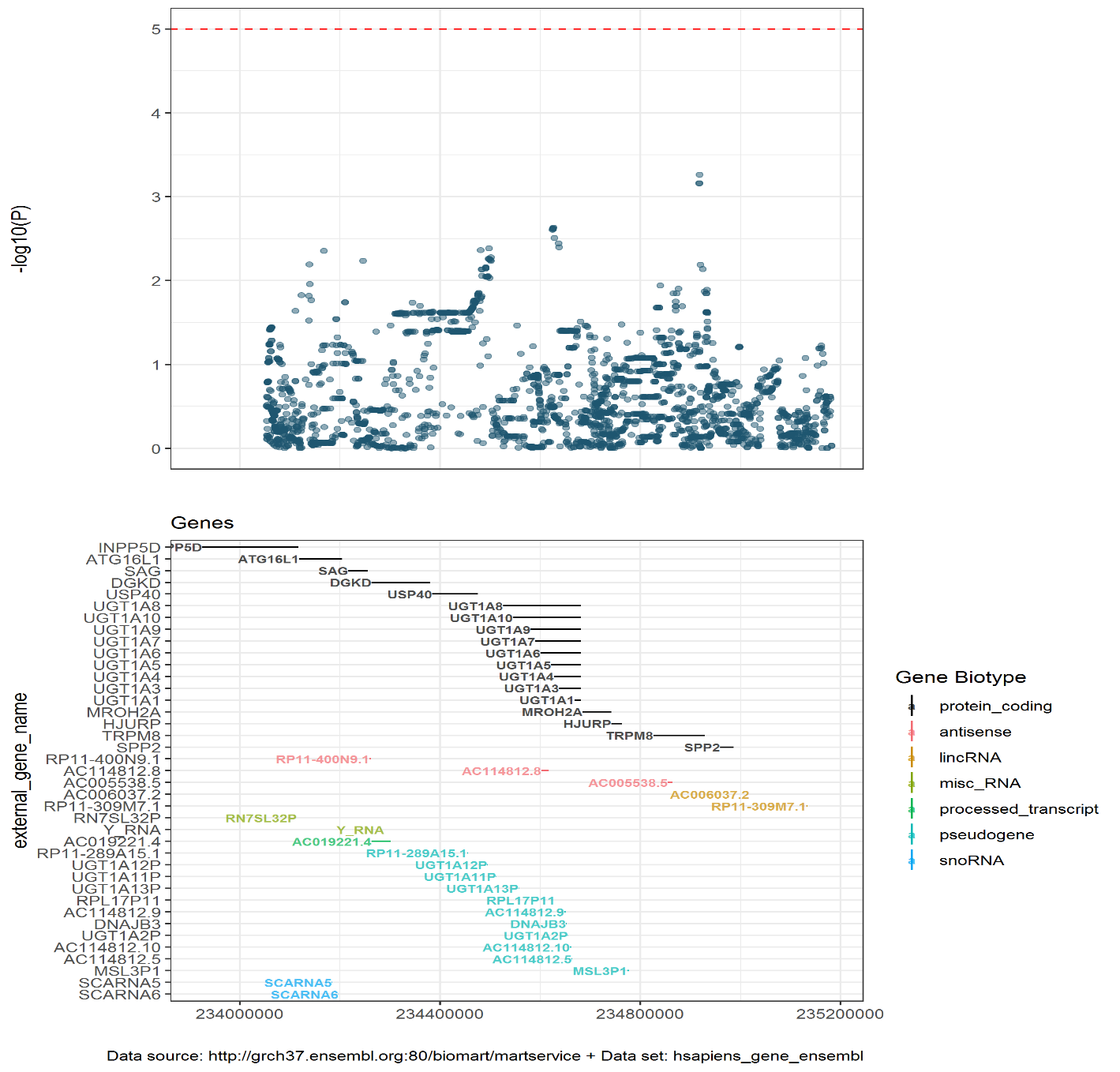
**

**Figure S5.　P value of *UGT1A8* region ± 500 Mb from response to raloxifene at LS site GWAS.**

**
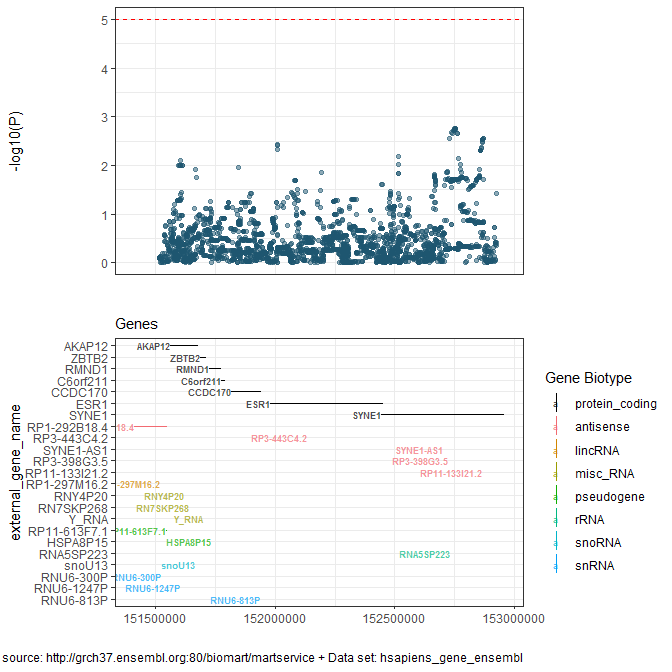
**

**Figure S6.　P value of ESR region ± 500 Mb from response to raloxifene at FN site GWAS.**

**
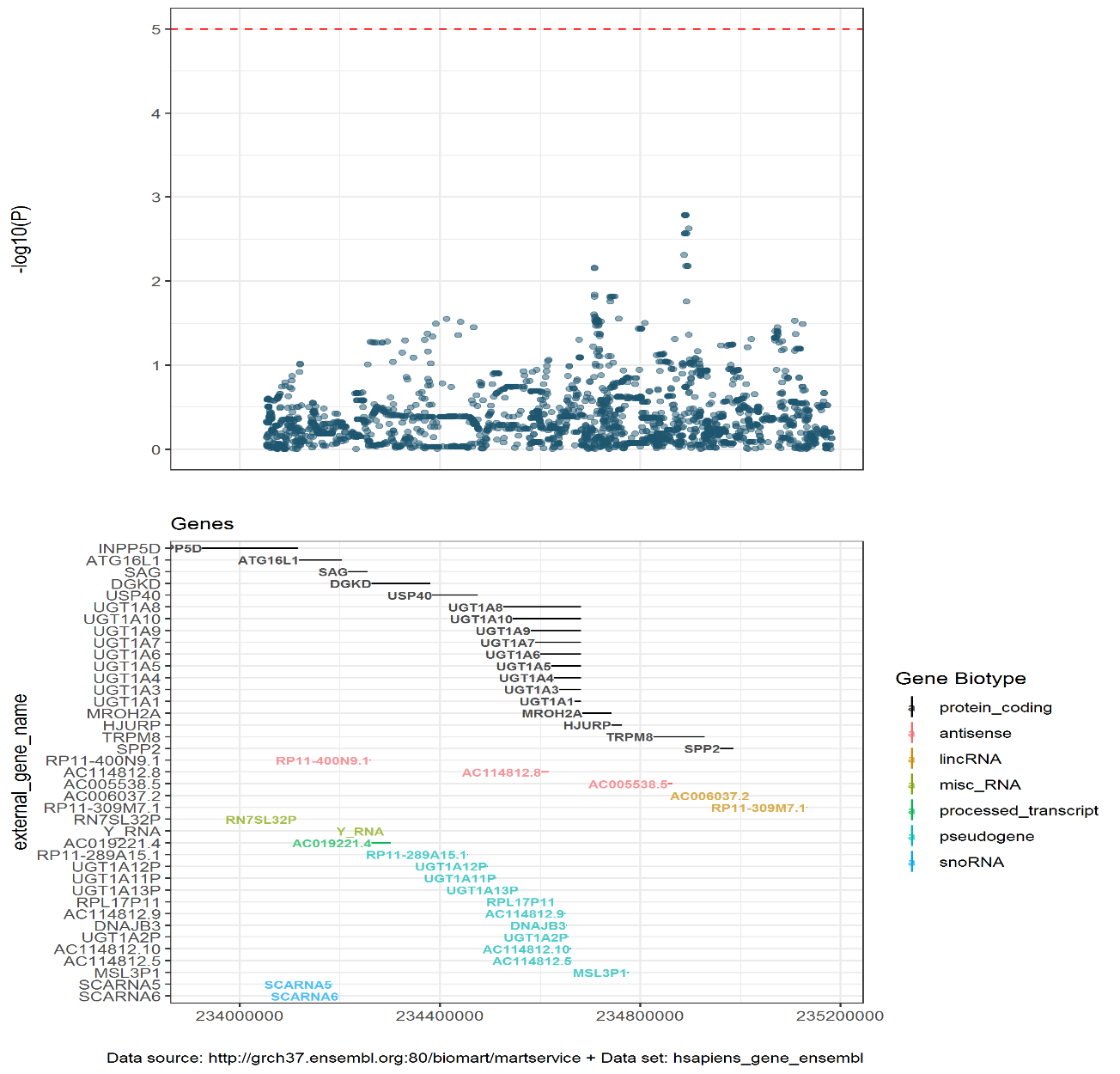
**

**Figure S7.　P value of *UGT1A8* region ± 500 Mb from response to raloxifene at FN site GWAS.**

**Suppl Tables.**

|  | Lumbar Spine | Femoral Neck |
| --- | --- | --- |
| Number | 34 | 41 |
| BMD change (%) | -0.938 ± 17.14 | -2.534 ± 15.846 |
| Follow up months | 34.65 ± 22.11 | 35.61 ± 23.54 |
| age | 71.09 ± 6.73 | 72.39 ± 6.99 |
| BMI | 23.39 ± 3.45 | 23.96 ± 3.49 |

**Table S1. Baseline characteristics of inclusion individuals.**

Data are presented as mean±SD.

**Table S2. Variants which reported to be related to raloxifene response previously**

|  |  | LS | | | | | |  | FN | | | | | |
| --- | --- | --- | --- | --- | --- | --- | --- | --- | --- | --- | --- | --- | --- | --- |
|  |  | A1 | A2 | MAF | BETA | SE | P |  | A1 | A2 | MAF | BETA | SE | P |
| *UGT1A8* | rs1042597 | C | G | 0.499 | -0.158 | 0.350 | 0.656 |  | C | G | 0.499 | 0.343 | 0.256 | 0.193 |
| *ESR1* | rs2234693 | C | T | 0.346 | 0.230 | 0.387 | 0.559 |  | C | T | 0.346 | -0.126 | 0.342 | 0.715 |
|  | rs9340799 | G | A | 0.192 | 0.089 | 0.608 | 0.885 |  | G | A | 0.192 | -0.064 | 0.525 | 0.903 |

LS, lumbar spine; FN, femoral neck

**Table S3. eQTL evidence of most significant SNPs.**

| Gene Symbol | SNP | P-Value | NES | Tissue |
| --- | --- | --- | --- | --- |
| *FUT9* | rs7768089 | 1.40E-14 | -0.66 | Pancreas |
|  |  | 2.50E-05 | -0.26 | Brain - Cerebellum |
| *CSRP2* | rs34311394 | 2.50E-13 | 0.79 | Brain - Putamen (basal ganglia) |
|  |  | 7.40E-13 | 0.54 | Brain - Nucleus accumbens (basal ganglia) |
|  |  | 5.50E-12 | 0.61 | Brain - Caudate (basal ganglia) |
| NES, normalized effect size | | | | |
